# Scaling up delivery of HIV services in Africa through harnessing trends across global emerging innovations

**DOI:** 10.3389/frhs.2023.1198008

**Published:** 2023-10-31

**Authors:** Moredreck Chibi, William Wasswa, Chipo Nancy Ngongoni, Frank Lule

**Affiliations:** ^1^ Science and Innovation, Assistant Regional Director, World Health Organization Africa Region, Brazzaville, Congo; ^2^ HIV, Tuberculosis and Hepatitis, Universal Health Coverage/Communicable and Non Communicable Disease Cluster, World Health Organization Africa Region, Brazzaville, Congo

**Keywords:** HIV services, devices, digital technologies, innovation, health systems strengthening

## Abstract

Globally, innovations for HIV response present exciting opportunities to enhance the impact and cost-effectiveness of any HIV program. However, countries especially in the African region are not on equal footing to effectively harness some of the existing innovations to accelerate impact on HIV services delivery. This paper aims to add to the discourse on innovative solutions to support countries to make informed decisions related to technologies that can be adapted in different contexts to strengthen HIV programs. A scoping review which involved a search of innovations that can be used in response to the HIV epidemic was carried out between June 2021 and December 2022. The results showed that a high level of technological advancement occurred in the area of digital technologies and devices. Out of the 202 innovations, 90% were digital technologies, of which 34% were data collection and analytics, 45% were mobile based applications, and 12% were social media interventions. Only 10% fell into the category of devices, of which 67% were rapid diagnostic tools (RDTs) and 19% were drone-based technologies among other innovative tools. The study noted that most of the innovations that scaled relied on a strong ICT infrastructure backbone. The scoping review presents an opportunity to assess trends, offer evidence, and outline gaps to drive the adoption and adaptation of such technologies in Africa.

## 1. Introduction

The burden of HIV continues to be a protracted developmental challenge for the African continent, with East and Southern Africa being the regions worst affected (1). The COVID-19 pandemic has been reported to have worsened the situation by instigating disruptions to HIV services (2). According to the report by the Joint United Nations Programme on HIV/AIDS (UNAIDS), the pandemic was something to be wary of, as a 6-month complete disruption in HIV treatment could have caused more than 500,000 additional deaths in sub-Saharan Africa between 2020 and 2021 (3). This would return the region to 2008 AIDS mortality levels (4). It is important to find innovative ways for interventions that circumvent the effect of disruptions like pandemics to improve or facilitate linkage to antiretroviral therapy (ART) care and retention of the practices and processes that are in place in low- and middle-income settings (5). Moreover, despite the increasing global coverage of HIV testing, treatment services, and enabling technologies, key populations such as drug users and sex workers are still underserved (6).

Given that there is a huge asymmetry to the information regarding access to technological innovations for HIV services among African countries, a proactive approach is desired to collate innovations deployed in other parts of the world and share with countries in the African region. This helps to facilitate informed decisions about the possibility of adopting and adapting such innovations in the region. This is a multistage process that aligns with scanning the technological landscape then assessing the various resources needed for successful and sustainable integrations and implementations. This paper addresses the first stage of understanding the various options available by collating innovations that support efficient HIV service delivery across the continuum of prevention, diagnostics, treatment and management, access to HIV services, behavioral change, and strategic information. The key question guiding the review is *What technologies are currently being used in the delivery of HIV services and what are the key factors for creating an enabling environment for the adoption of these technologies in the African context?* The article proposes that assessing such technologies helps facilitators and HIV program coordinators be proactive in resource mobilization and creating enabling environments for successful integration of these innovations. Moreover, this helps in adjusting to the new normal of blended services along all aspects of service provision in the healthcare sector.

To ensure alignment, this paper will be based on the following definitions. Innovation is noted as new (re)combinations of ideas as outlined by Schumpeter (7). This innovation can encompass product, process, or systemic innovations introduced into the health systems in an incremental, radical, or disruptive manner (8, 9). In particular, innovation in healthcare has been defined as a novel idea or set of behaviors, routines, and/or ways of working that involve a change in practice within a healthcare setting (10). As Peter Drucker highlighted, this innovation can be intrinsic or extrinsic to the system or organization (11). Technology is defined as a practical application of scientific knowledge and discoveries for the purpose of creating tools, systems, and methods that simplify health system workflows^1^. This can be systemic in how messages are conveyed or in the form of a tangible device.

The remainder of this article is structured as follows: Section 2 presents the summarized outline of other research related to technological usage in the delivery of HIV services. Section 3 describes the methods utilized in this study and the results are presented in Section 4. Section 5 presents the policy and technological implications of the various findings from the study.

## 2. Technology and HIV services

Technology usage in HIV services is one key aspect that has gained momentum over the past 10 years with the intent of scaling HIV prevention efforts (12). The World Health Organization (WHO) developed guidelines on leveraging technologies, innovation, and digital solutions for HIV prevention, diagnosis, treatment, and care for key populations (13). This is especially around consideration of the underlying socio-cultural, economic, political, legal, and various other contextual factors. Muessig et al. (14) looked at how technologies could be applied at various stages of HIV care across primary and secondary prevention activities in the period of 2013–2014. The tools that the authors covered included social networking sites, provision of real-time assessment and feedback, gamification, and virtual reality. These are all key aspects which can be utilized especially in this era of COVID-19. They identified that gaps remain around linkage to care, retention in care, and initiation of antiretroviral therapy, and this was pre-COVID. Notably, they only focused on completed and planned interventions conducted within the United States (14).

**Figure 1:**
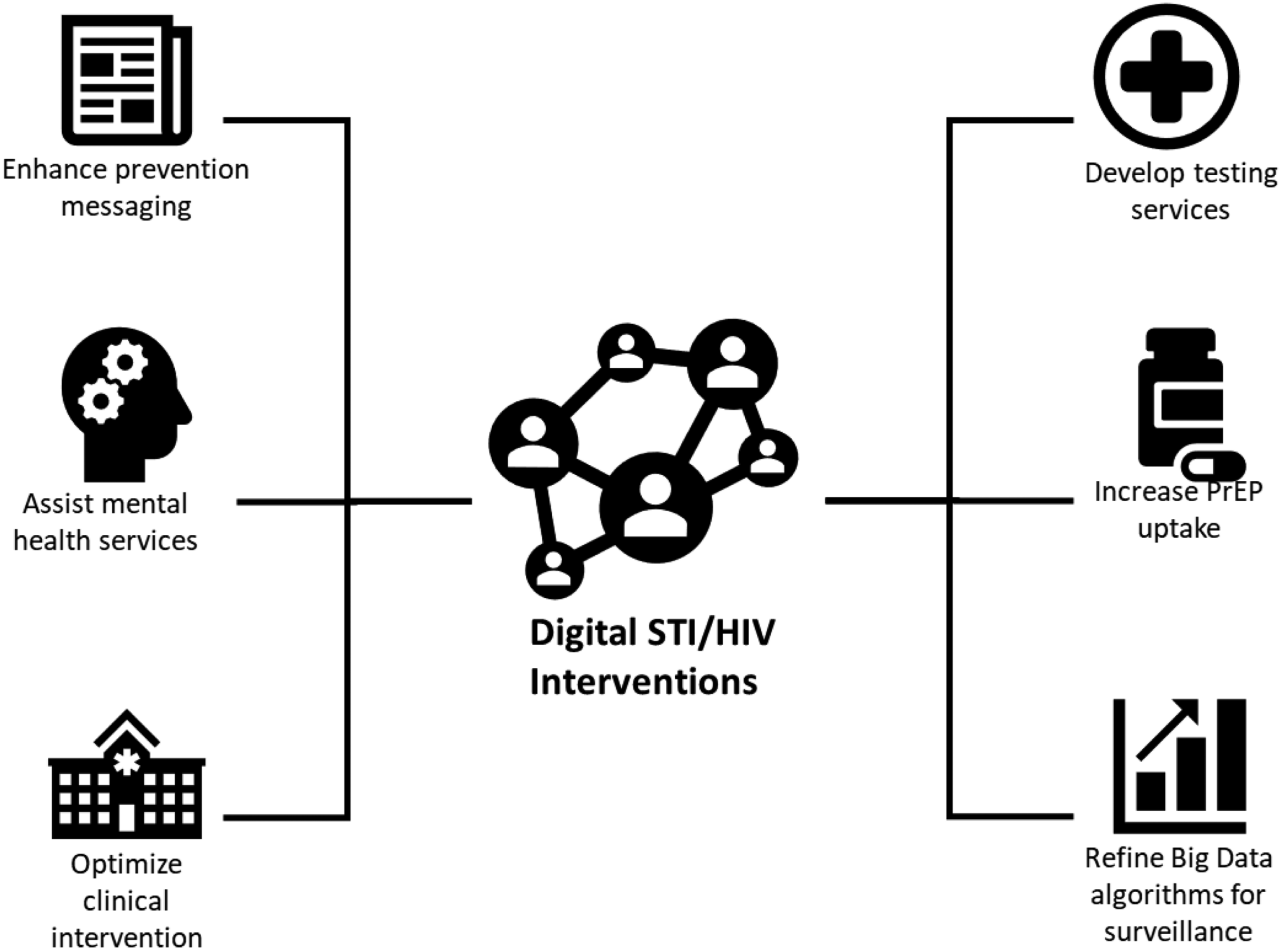
Type of digital STI/HIV interventions. Source: Cao et al. (16).

Another study by Navarra et al. (15) looked at the US trends for technology-enabled adherence interventions among HIV-infected youth between the ages 13 and 29 years. The aim of this study was to look at the feasibility of computer-based interventions and the efficacy of SMS texting for adherence support among HIV-infected adolescents and young adults. Cao et al. (16) did a scoping review of 23 studies between March 2018 and August 2019 where the studies looked at how digital technologies can be utilized to enhance prevention messaging, develop testing services, increase pre-exposure prophylaxis (PrEP) uptake, refine big data algorithms for surveillance, optimize clinical intervention, and assist mental health services. They highlighted the need to consider strategic implementations that leverage digital platforms for network-based interventions and evaluate their roles on a community level. More importantly, they gave a solid categorization of the types of digital HIV interventions shown in Figure 1. The aspects in that paper around issues such as prevention, mental health, and big data can help outline the monitoring and evaluation aspects that direct the norms of individual digital intervention platforms for HIV services.

On the other hand, Horvath et al. (17) highlighted how technology-assisted HIV testing interventions are strategically important to reach national and global targets for HIV status awareness. They put forward those questions that are still being asked around ascertaining which interventions work for various target populations, how technology like social media platforms can be leveraged to promote HIV testing services, and best practices for scaling up mobile heath (mHealth) and other technology-based interventions (17). Interestingly, another study by Veronese et al. (18) showed that over 50% of HIV testing uptake occurred due to exposure to digital communication interventions. The review compared the impact of generic and less interactive online messaging to various tailor-made interventions using online social networking and online engagement tools such as videos and digital messages (18). Notably, tracking and synthesizing technology tools such as mHealth innovations in HIV is a bit difficult due to the broad range of tools and initiatives and speed of innovation (19). This comes from a rapidly changing pipeline of technology-based care and prevention methods and to assess whether the interventions are appropriately diversified (20).

There are important technology and regulatory gaps in low- and middle-income countries with a need for further development of post market surveillance systems for HIV-related services (21). An additional call has been made for researchers to invest in more efficient and expedited intervention development so that current and future needs are addressed (20). The works described above all looked at the important facets of impact of technology on HIV services; however, there still seems to be a void in understanding and visibility of what types of technologies are being utilized for HIV services. This paper aims to raise awareness of the trends and hence be part of foundational discussions on the various technologies that are available for HIV services that can benefit other contextual settings especially in Africa.

## 3. Methodology and approach

### 3.1. Data sources, search strategy, and study selection

This study adopted a systematized search strategy to identify innovations across five scientific databases (PubMed, Google Scholar, Scopus, IEEE, and Science Direct) using different keywords pertaining to different HIV response areas, i.e., HIV prevention, diagnosis, treatment and management, strategic information, access to services, and behavioral change were combined with the words “innovations or technology” in the search query. Duplicates, drugs, vaccines, and social programs were excluded. A total of 180 papers were identified; however, after screening, only 38 papers were included.

Additionally, innovations were found across gray sources such as the Google Play Store, iOS App Store, and websites. The search for mobile apps was conducted using the same set of keywords pertaining to the HIV response area. The applications had to have a description and have 1,000 + downloads to be included in the analysis and a total of 418 apps were identified and 25 were included in the analysis. Apps not in English were excluded. From websites, the web content mining encompassed technologies published on different cooperate organizational websites, social media channels like Twitter, and legitimate news websites, which resulted in the identification of 139 innovations. Innovations that did not have functional and tested prototypes and were not related to HIV were excluded.

**Figure 2:**
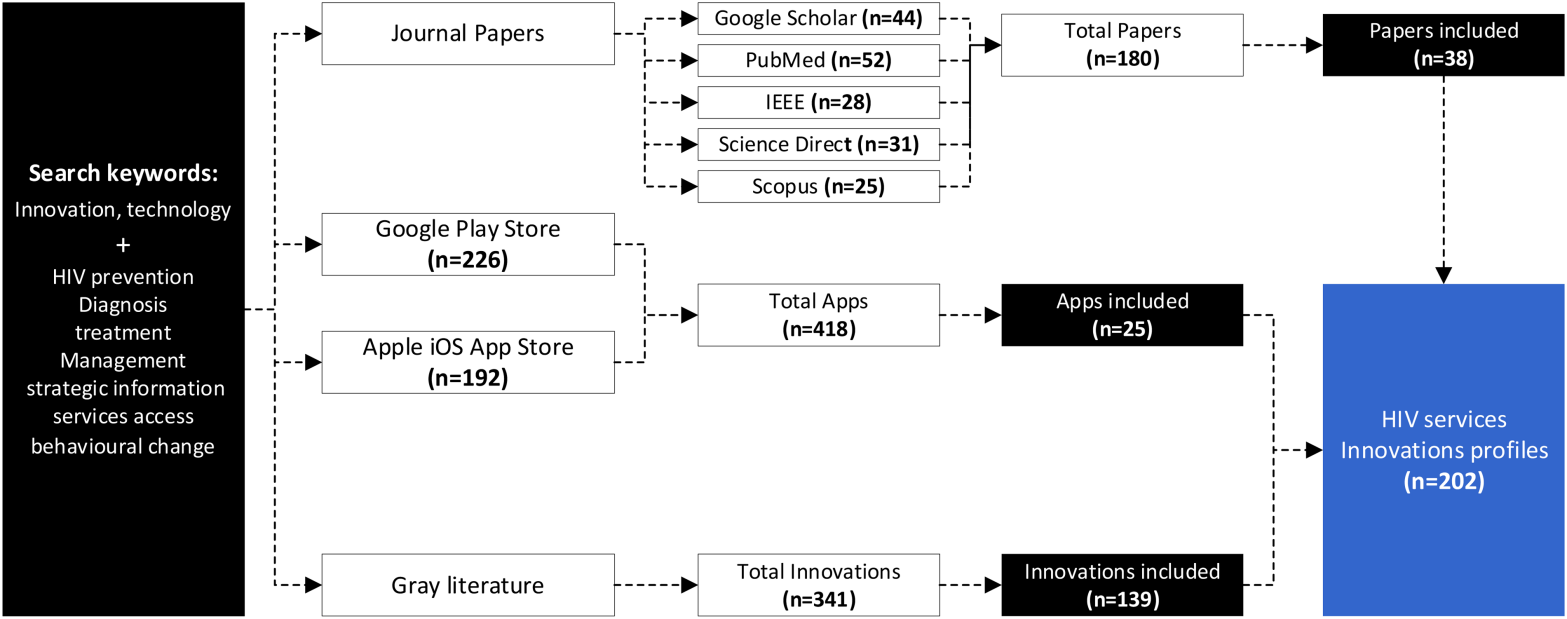
The HIV services innovations search strategy.

Overall, a total of 202 HIV innovations (38 from scientific databases, 25 from the Google Play and iOS App stores, and 139 from web-content mining) were considered for detailed analysis. The process is shown in Figure 2.

### 3.2. Data analysis

**Figure 3:**
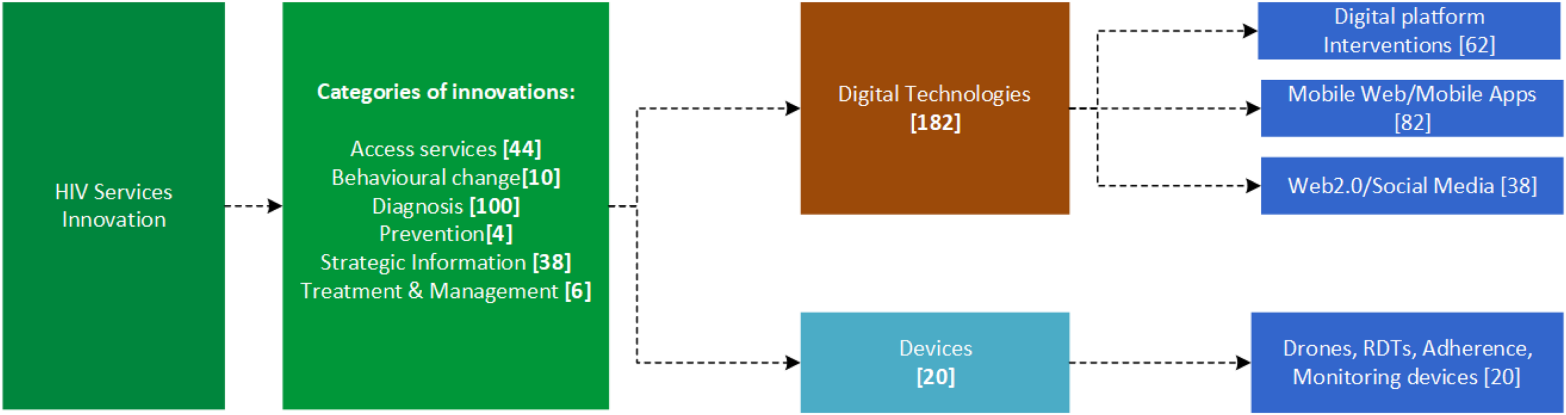
The HIV services technological innovations categories.

The identified innovations were profiled using the name of the innovation, company or organization, country of origin, description of the innovation, the category assigned to the innovation, and the URL link to indicate the source. The innovations were categorized according to intervention areas, which included prevention, treatment, services access, diagnostics, behavioral change, and strategic information, and classified as a digital technology (i.e., digital platform-based interventions, mobile web/mobile apps, or Web 2.0/social media) or as a device [i.e., drones, rapid diagnostic tools (RDTs), adherence monitoring devices, etc.], as shown in Figure 3.

## 4. Findings and key observations

The study revealed that a myriad of innovations has been developed across the world to contribute to equitable access to HIV services, with the majority of them emerging from countries like the United States of America, Switzerland, and China. Out of the 202 HIV services innovations, 90% were digital technologies whilst 10% were devices. Under the digital technologies, the split was 34% being web-based innovations, 45% mobile based applications, and 11% Web 2.0/social media interventions. On the other hand, in the devices category, 67% were RDTs, 19% drone-based technologies, and 14% were other tools developed specifically for HIV services. The following sections will highlight some of the key selected digital initiatives and devices that have been rolled out in various settings to accelerate scaling up access and utilization of HIV services and products. These innovations include therapeutic products like microbicides (22), post-exposure prophylaxis (23), and pre-exposure prophylaxis (24).

### 4.1. Digital platform-based interventions used in HIV services

The analysis showed that web-based technologies around the creation of digital platforms are increasingly being used in HIV service delivery across the continuum of prevention, diagnostics, treatment, access to HIV services, behavioral change, and strategic information. For example, the Open Medical Record System (OpenMRS)^2^ has been widely used as an electronic medical record storage and retrieval system for managing millions of HIV/AIDS and tuberculosis (TB) patients in the developing world. OpenMRS has been used and evaluated in different countries like Rwanda, Haiti, Lesotho, Mozambique, Pakistan, Philippines, Rwanda, South Africa, Tanzania, Uganda, and Zimbabwe (25). Allen et al. (26) reported the experience in implementing the OpenMRS medical record system to support HIV treatment in Rwanda. They found out that OpenMRS addresses the problem of configuring electronic management records (EMR) systems to suit new sites, languages, and diseases (26). Other medical record systems have been developed inspired by the OpenMRS architecture. Tweya et al. (27) in Malawi developed a point-of-care electronic medical record system for TB/HIV co-infected patients and evaluated it in a public clinic in Malawi that serves HIV- and TB-infected patients. Their experience suggests that an electronic medical records system can improve patient management, enhance integration of TB/HIV services, and improve provider decision-making (27). Haskew et al. (28) implemented a cloud-based electronic medical record to reduce gaps in the HIV treatment continuum in rural Kenya. Implementation of the EMR system significantly improved data quality and provision of clinical care, helping to ensure patients who are eligible for HIV treatment receive it early (28).

The Therapeutic Education System (TES)^3^ developed in the USA is an interactive, web-based program theoretically grounded in the evidence-based community reinforcement approach to behavior therapy (29). The TES is composed of interactive, multimedia modules, including those focused on cognitive behavioral skills training. The efficiency of therapeutic education systems has been evaluated by several authors (30). A comparative study of the TES and standard treatment by Chaple et al. (31) showed that the TES and standard treatment were equally effective in reducing criminality, relapse to drug use, and HIV risk behavior. TES solutions are offered by several service providers including Total Education Solutions^4^ and the Center for Technology and Behavioral Health^5^.

The HIV DHIS2 packages and tools developed by the Global Health Infrastructures Group at the University of Oslo in partnership with the World Health Organization (WHO) support HIV case surveillance tracking and aggregation. BAO Systems teamed with FHI 360 and others to develop a standardized tracker metadata package called the DHIS2 HIV Case Surveillance Package^6^. It facilitates automated reporting of 70+ President's Emergency Plan for AIDS Relief (PEPFAR) monitoring, evaluation, and reporting and custom indicators, while allowing flexibility to customize the model to meet local reporting requirements. DHIS2 has been used by organizations such as MEASURE Evaluation^7^ to develop comprehensive guidance for developing an electronic solution to track patients across the prevention of mother-to-child transmission (PMTCT) of the HIV continuum of care, with several positive results reported by different authors (32). DHIS2 has also been used to generate HIV-indicator data for decision-making in Kenya (33). However, prior to the adoption of DHIS2 by some countries, there existed other systems, for example, in Kenya, the Kenya HIV/AIDS Program Monitoring System (KePMs). KePMs is a computerized database for the management and analysis of the PEPFAR, treatment, and prevention indicators required by the United States of America Government program managers. The evaluation of this system showed a consistency rate of 79.5% with the DHIS2 system (34).

In Mozambique^8^, the Drug Resources Enhancement against AIDS and Malnutrition (DREAM)^9^ software was born out of the need to computerize the management of the clinical files of the project patients. The DREAM software is now being scaled up in Angola, Cameroon, Democratic Republic of Congo, Guinea, Kenya, Malawi, Nigeria, Eswatini, and Tanzania. The DREAM software has also been considered an option as a tool to be used in the implementation for the prevention of mother-to-child transmission in Cameroon (35).

Web-based systems have also been used in supporting access to services. In Thailand, Adam's Love, a novel online-to-offline (O2O) model, was implemented for disseminating HIV education and counselling (36). The tool's online-to-offline model helps in identifying, reaching, and linking vulnerable populations to HIV clinical services. The Adam's Love (O2O) model has been reported to be highly effective in linking at-risk individuals from the LGBTQ + community to PrEP and HIV testing services. This tool has high potential to be replicated and scaled up in other settings with erratic power supplies and high internet penetration rates among key populations (36).

Web-based tools also make it possible to integrate data from different sources. In Haiti, a national HIV reporting electronic platform known as Suivi Actif Longitudinal du VIH en Haiti (SALVH)^10^ is being used to integrate data from multiple sources into a single national dataset. The platform makes it possible for authorized users to analyze and visualize custom reports and dashboards from the national SALVH database. The tool has been found to be very effective in providing real-time aggregated national data for strategic planning (37). In Botswana, the Botswana Harvard AIDS Institute Partnership developed the Botswana Combination Prevention Project (BCPP) data linkage tool^11^ to support individual-level patient tracking. In New York, Healthix,^12^ the largest public health information exchange (HIE) in the USA sends alerts and offers Consolidated Clinical Document Architecture (C-CDAs) to providers in real time when an HIV or AIDS patients needs medical attention. The Human Resource for Health (HRH) inventory tool provides countries with a wealth of information about donor investments in HRH (38). By profiling donor investments, development partners and host governments can more easily track and analyze investments in HRH staffing, down to the site level, which can be utilized for more robust sector-wide performance monitoring and HIV services program planning.

### 4.2. Mobile-based interventions used in HIV services

The analysis revealed numerous mobile based solutions that help people living with HIV to adhere to medication schedules, improve access to services for patients, and assist administrators to have access to strategic information. The Klick app developed by ViiV Healthcare in Europe uses digital tools to triage HIV patients according to clinical needs (39). The app allows patients to manage appointments, complete health assessments, review results, and communicate with their healthcare team. The WHO HTS Info App^13^ provides on-the-go access to WHO's current HIV testing services guidelines and information, whilst the WHO HIV Tx App^14^ offers consolidated guidelines and documents for HIV treatment and care. The national prevention information network (NPIN) PrEP provider data and locator widget^15^ was developed to enable people in the USA to search for PrEP providers according to their zip code, state, or full address, and filter out services that do not offer PrEP to the uninsured. The HIV iChart app^16^ provides up-to-date information on potential reactions between antiretroviral drugs and other prescription drugs, as well as over the counter, recreational, and alternative medications. SmartLink was a mobile phone app developed in South Africa which could provide HIV patients with laboratory test results and be used for providing care and information after diagnosis (40), whilst PositiveLinks is an app that was designed to deliver just-in-time messaging with the aim to improve linkage and retention among people living with HIV (41).

The use of digital games is rapidly becoming an important tool for improving health behaviors and supporting the delivery of care and education especially among HIV patients (42). As a result, gamification has been found to be an effective way to reach certain groups with information about HIV prevention, testing, and treatment services, particularly young people (43). Tumaini, developed by the Centre for Global Health Research in Kenya, is a smartphone game-based intervention that prevents HIV among young Africans (44). It is designed to increase the usage of condoms by increasing knowledge about sexual health and HIV. Evaluation of Tumaini in Kenya showed that the intervention arm showed significant gains in sexual health-related knowledge and self-efficacy, behavioral intention for risk-avoidance strategies, and sexual risk communication compared with the control arm (44). The National AIDS Control Organization (NACO) AIDS^17^ app is used to raise awareness through its gamification features.

P3 (Prepared, Protected, Empowered)^18^ developed in the USA is a smartphone app for HIV-uninfected young men who have sex with men and young transgender women who have sex with men that utilizes social networking and game-based mechanics to improve PrEP adherence. In Eswatini, SwaziYolo^19^ is an interactive game for HIV-negative young adults living in Eswatini. In this app, players can practice relationship and health choices while looking for love. UNESCO developed Fast Car: Travelling Safely around the World (45), which is a racing game that helps you to learn about HIV and AIDS prevention. The game aims to provide young people with accurate and reliable information about HIV prevention, intending to educate and entertain as well as promoting healthy behavior.

A myriad of HIV-related applications has also been developed to support HIV diagnosis and access HIV services. HIVSmart^20^ was developed by a team of scientists and physicians at the Research Institute of the McGill University Health Centre to assess users’ HIV exposure risk, interpret their self-test results, then link those who test positive to treatment. The HIV self-testing (HIVST) mobile application (app) was developed for use in South Africa to reduce barriers associated with facility-based testing (46). The Doctor On Demand app^21^ allows users to access different health specialists to get health advice, basic medical exams, diagnoses, prescriptions, and other medical care quickly, whilst the GoodRx^22^ helps users locate pharmacies, compare prices, and find ones with the lowest cost. Apps like Life4me+^23^, Daily Charge^24^, and the HIV Atlas app^25^ push notifications and reminders to users about appointments and medications as well as track lab results. An interesting design is a the aDOT platform which is integrated with a mobile app (LYNX) designed to support HIV testing and PrEP uptake designed by Liu et al. (47).

Additionally, apps have been developed to provide social support to those living with HIV through communities. myHIVteam^26^ is a social network for anyone living with HIV whilst Positive Peers^27^ is a private support app for young people living with HIV. There are also several dating apps like Positive Singles, Positive Match, and Hzone that are connecting people with STDs, including HIV. It is key to note that consideration of resource-constrained areas is also what pushes mobile-based services. The use of unstructured supplementary service data (USSD) and short message services (SMS) to send reminders or information is one key feature across mobile apps.

### 4.3. Social media-based interventions used in HIV services

Social media platforms play a key role in the mental wellbeing and support of HIV services delivery. Some organizations are using social media to target and interact with people living with, most affected by, or at risk of HIV who may feel isolated or less visible. Organizations like Avert^28^ create campaigns to increase the awareness and understanding of HIV and sexual health around the world. These campaigns include the #StandUpToHIV empowers people to get over their fears of what happens during a test and what other people will think, and #KnowTheScore which aims to get young men in Southern Africa tested for HIV. HIV social media kits^29^, which are a collection of key messages, graphics, and sample content shared on social media that strengthen communications for national HIV awareness days, have been developed by several organizations such as the Centers for Disease Control and Prevention (CDC) and HIV.gov^30^ to support consistent communication across organizations. These collection of social media toolkits can be used to promote HIV prevention, testing, treatment, and anti-stigma messaging to social media audiences by other organizations.

Chatbots have also been widely used in HIV services with ongoing research in improving the interactivity of the chatbots to make conversations more natural. Ardiana et al. (48) built a mobile-based chatbot application using artificial intelligence markup language (AIML) to aid the foundation in giving people reliable HIV/AIDS related information. AIML was used because it can make bots closer to human capabilities with the aim of emulating human conversation as good as a counseling session. Other notable chatbots include the Marvin chatbot, developed by McGill University which uses AI to engage HIV patients in their antiretroviral therapy (49); the Eli chatbot developed by the United Nations Educational Scientific and Cultural Organization (UNESCO) Institute for Information Technologies in Education (IITE) and which can answer questions about growing up, love, relationships, and sexual health, including HIV prevention and treatment (50); SHIHbot is a Facebook chatbot for sexual health information on HIV/AIDS (51); and Sophie Bot^31^, developed in Kenya, also offers information on reproductive health. Additionally, these chatbots are being integrated into communications apps such as WhatsApp, Telegram, and Signal, helping to disseminate information and connect citizens with trained healthcare providers and counsellors at times and in places that suit them^32^.

### 4.4. Highlights of devices used in HIV services

The devices presented in this paper have been categorized into adherence monitoring devices, drones, and digital test kits. There are three types of HIV diagnostic tests: nucleic acid tests (NAT), antigen tests, and antibody tests (52). Blood tests are the most common way to diagnose HIV. However, over the years, other non-invasive body fluid-based tests have been developed. These include the oral fluids-based tests like those that use saliva and urine as samples (53).

Several innovative rapid test kits (RDTs) have been designed that enable home-based HIV testing. These include the SURE CHECK® HIV 1/2 ASSAY^33^, a self-test for the detection of antibodies to Human Immunodeficiency Virus Types 1 and 2 (HIV 1/2) in fingerstick whole blood. The SURE CHECK HIV Self-Test is intended for use by untrained users to aid in the diagnosis of HIV infection. Other self-test kits include the Mylan HIV Self-Test^34^ and INSTI® HIV Self-Test^35^. Over the years, non-invasive test kits have been developed that do not require blood and these include DPP HIV 1/2 Assay^36^, EXACTO©^37^ TEST, and OraQuick HIV Self-Test^38^ that use an oral swab for testing. There are also innovations that identify and determine the percentages and absolute counts of mature human T-lymphocytes (CD3+) and helper/inducer (CD3 + CD4+) T-lymphocyte subsets in erythrocyte-lysed blood such as the PIMA CD4 Test (54). These are used to monitor and evaluate the body's response to ART.

Due to the increasing processing power of mobile devices, new technologies harnessing the potential of mobile phone sensors, cameras, processing power, and data sharing capabilities have been developed to improve HIV diagnosis. Mobile-based apps have been developed that can read test results from an image taken by end users on a mobile device. These apps can automatically analyze the images and are also able to report results to public health systems for better data collection; for example, a pioneering technology developed by University College of London (UCL) and Africa Health Research Institute (AHRI) researchers could transform the ability to accurately interpret HIV test results, particularly in low- and middle-income countries (55). This is based on AI image analysis for the diagnosis of HIV from test images. Several reports and reviews have highlighted the potential of AI in HIV services especially for instant diagnosis; however, few apps have been clinically tested and deployed for clinical use (56).

Electronic adherence monitors (EAM) are another category of devices that have been greatly adopted in HIV services. These monitors include the medication event monitoring system (MEMS) adherence monitor^39^ that processes patient data using predefined and validated algorithms to present a comprehensive picture of patient adherence based on the dosing history from the MEMS packages. It collects adherence data from digitally enabled hardware, through sensors and stores it in a database. The Wisepill RT2000 Medication Dispenser^40^ is an internet-enabled medication dispenser allowing remote, real-time medication management. The rich feature set makes it a favorite for adherence research and clinical trials. Other adherence devices include the Wisebag (57), AdhereTech^41^, MedMinder ^42^, and Tell-Me-Box^43^.

Drone technology has also been used in HIV services especially for circumvent supply chain disparities in the delivery of HIV drugs. Drones have been used in Uganda to carry HIV medicines^44^ to isolated community groups and health facilities. These have also been used in also other countries like Malawi to improve the testing labs’ footprints from the central laboratory to the health facilities.^45^

## 5. Discussion and conclusion

In the study analysis, more than 75% of the innovations were developed and deployed in Western and Asian countries including the United States of America, Switzerland, and China, with very few from Africa. Innovation output is still relatively low across the African continent as depicted in the 2022 edition of the Global Innovation Index (58).

Dionne (59) highlighted how interventions in HIV/AIDS often encompass global actors and argued that misaligned priorities usually can create multiple opportunities for failure. Hence, this study sought to decrease the failure rate by unlocking the potential for mapped innovations where African governments should reflect on how such innovations can be integrated to improve healthcare service delivery while taking ownership. The paper offers visibility of technologies that address various aspects of HIV response, making it a starting point which organizations such as the World Health Organization (WHO) and other implementing partners may use as a source of innovations to foster public–private partnerships and initiate strategic dialogue in developing countries to pave a way forward to localization of these technologies. It is important to note that co-creation is of paramount importance as exemplified in Southwestern Uganda, where the information and mass media campaigns aimed at improving adherence to ART among adolescents was designed to cater for the needs of the adolescents through consultations (60).

As countries are exploiting the opportunities for adopting and adapting these innovations in their respective contexts, they should take into consideration aspects to do with: (a) *Privacy and security*, where a lot of content that is stored on platforms where users enter their information needs to be protected and also offer the users some form of data protection; *(b) inclusive innovation,* where key stakeholders are involved in tool selection or development. This helps standardization of the HIV services to increase trust, acceptance, and uptake of the tools for HIV service delivery; (c) *technical capacity and skills,* where training is needed to pass on the knowledge and skills required to design and maintain the technological infrastructure; and (d) *policy and infrastructure,* where even though the African region is doing a lot of work aligned with several strategies, policies and frameworks that are looking at how best to integrate telemedicine, AI, big data, and drones in health systems strengthening, there are still more opportunities for leapfrogging improvements in the delivery of HIV programs.

Considering some limitations of this paper, there is a lack of longitudinal analysis of some of the innovations to track and trace how best they can be integrated into strengthening health systems in African countries. Researchers and implementers should undertake longitudinal studies that further investigate the cost-effectiveness of the technologies that have been integrated into health systems. It is important to undertake a cost–benefit analysis to see if the innovations actually improve the cost effectiveness of HIV programs and how they are implemented. Integrating healthcare interventions and innovations is initially a costly and time-consuming process, hence it is important to ascertain if the benefit that is purported translates to impact.

Overall, there is a big technological shift with the use of big data, artificial intelligence, and other emerging technologies to support improvements in the delivery of HIV programs. It is therefore critically important for countries in Africa to initiate internal dialogues on how best to exploit these emerging technologies to accelerate health impact. Furthermore, the findings of this analysis of the HIV services innovations can help in providing implementation strategies to African countries on how to adopt some of the technological innovations.

## Footnotes

^1^ https://resorcio.com/categories/technology-1628491222410

^2^ https://openmrs.org/

^3^ http://sudtech.org/about/

^4^ https://www.tesidea.com/

^5^ https://www.c4tbh.org/program-review/therapeutic-education-system-tes-2/

^6^ https://dhis2.org/metadata-package-downloads#hiv-tracker

^7^ https://www.measureevaluation.org/resources/pmtct-tracker.html

^8^ https://www.hrhresourcecenter.org/node/561.html

^9^ https://www.dreamprog.org/whatisdream/

^10^ https://salvh.mesi.ht/pages/Default.aspx

^11^ https://github.com/botswana-harvard

^12^ https://healthix.org/

^13^ https://play.google.com/store/apps/details?id=com.whohtsinfo&hl=en&gl=US

^14^ https://play.google.com/store/apps/details?id=uk.co.adappt.whoarv&hl=en&gl=US

^15^ https://npin.cdc.gov/preplocator

^16^ https://play.google.com/store/apps/details?id=com.liverpooluni.icharthiv&hl=en&gl=US

^17^ https://play.google.com/store/apps/details?id=com.naco.nhp&hl=en&gl=US

^18^ https://batlab.web.unc.edu/p3/

^19^ https://www.formula-d.com/projects/hiv-prevention-game/

^20^ https://hivsmart.org/

^21^ https://www.doctorondemand.com/

^22^ https://www.goodrx.com/

^23^ https://life4me.plus/en/about-us/

^24^ https://play.google.com/store/apps/details?id=com.gilead.dailycharge&hl=en&gl=US

^25^ https://m.apkpure.com/hiv-atlas/com.halosys.hivatlas.main

^26^ https://play.google.com/store/apps/details?id=com.myhealthteams.myhivteam&hl=en&gl=US

^27^ https://positivepeers.org/

^28^ https://www.avert.org/who-we-are

^29^ https://www.cdc.gov/stophivtogether/partnerships/social-media-toolkit.html

^30^ https://www.hiv.gov/

^31^ https://www.facebook.com/misssophiebot/

^32^ https://breakthroughactionandresearch.org/regional-interactive-fp-rh-information-center/

^33^ https://www.who.int/diagnostics_laboratory/evaluations/pq-list/191129_pqdx_0054_006_01_sure_check_hiv_self_test.pdf?ua=1

^34^ https://www.mylanez2uz.com/

^35^ https://www.insti.com/hiv-self-test/

^36^ https://www.who.int/diagnostics_laboratory/evaluations/pq-list/hiv-rdts/160606PQPR_0053-006-00_DPPHIV_v2.pdf

^37^ https://www.aidsmap.com/about-hiv/how-accurate-self-testing-hiv

^38^ https://www.rockethealth.shop/product/oraquick-hiv-self-test/

^39^ https://www.aardexgroup.com/solutions/mems-adherence-software/b

^40^ https://www.wisepill.com/

^41^ https://www.adheretech.com/

^42^ https://www.medminder.com/

^43^ https://clinicaltrials.gov/ct2/show/NCT03086655

^44^ https://theacademy.co.ug/medical-drones-project/

^45^ https://www.unicef.org/malawi/reports/drones-factsheet
